# Combining Genetic and Demographic Data for the Conservation of a Mediterranean Marine Habitat-Forming Species

**DOI:** 10.1371/journal.pone.0119585

**Published:** 2015-03-16

**Authors:** Rosana Arizmendi-Mejía, Cristina Linares, Joaquim Garrabou, Agostinho Antunes, Enric Ballesteros, Emma Cebrian, David Díaz, Jean-Baptiste Ledoux

**Affiliations:** 1 Departament d´Ecologia, Facultat de Biologia, Universitat de Barcelona, Avinguda Diagonal 643, 08028, Barcelona, Spain; 2 Institut de Ciències del Mar (ICM-CSIC), Passeig Marítim de la Barceloneta 37-49, 08003, Barcelona, Spain; 3 CIMAR/CIIMAR, Centro Interdisciplinar de Investigação Marinha e Ambiental, Universidade do Porto, Rua dos Bragas 177, 4050-123, Porto, Portugal; 4 Departamento de Biologia, Faculdade de Ciências, Universidade do Porto, Rua do Campo Alegre, 4169-007, Porto, Portugal; 5 Centre d’Estudis Avançats de Blanes (CSIC), Accés Cala St. Francesc 14, 17300, Blanes, Spain; 6 Instituto Español de Oceanografia, C/ Moll de Ponent s/n, 07015, Palma de Mallorca, Spain; Duke University Marine Laboratory, UNITED STATES

## Abstract

The integration of ecological and evolutionary data is highly valuable for conservation planning. However, it has been rarely used in the marine realm, where the adequate design of marine protected areas (MPAs) is urgently needed. Here, we examined the interacting processes underlying the patterns of genetic structure and demographic strucuture of a highly vulnerable Mediterranean habitat-forming species (i.e. *Paramuricea clavata* (Risso, 1826)), with particular emphasis on the processes of contemporary dispersal, genetic drift, and colonization of a new population. Isolation by distance and genetic discontinuities were found, and three genetic clusters were detected; each submitted to variations in the relative impact of drift and gene flow. No founder effect was found in the new population. The interplay of ecology and evolution revealed that drift is strongly impacting the smallest, most isolated populations, where partial mortality of individuals was highest. Moreover, the eco-evolutionary analyses entailed important conservation implications for *P*. *clavata*. Our study supports the inclusion of habitat-forming organisms in the design of MPAs and highlights the need to account for genetic drift in the development of MPAs. Moreover, it reinforces the importance of integrating genetic and demographic data in marine conservation.

## Introduction

Marine protected areas (MPAs) are fundamental tools for the conservation of marine biodiversity [1]. However, their design has mainly focused on the conservation of economically important fish stocks (e.g. [2, 3]), while the maximization of biodiversity has been somewhat disregarded [4]. Habitat-forming (structural) organisms play a fundamental ecological role, as they act as ecosystem engineers and significantly increase the levels of biodiversity of the associated communities [5, 6]. In order to improve MPAs design and effectiveness in protecting biodiversity, the importance of including habitat-forming species in marine conservation planning has been recently underlined [7, 8].

Homogenizing genetic connectivity and disruptive genetic drift are two of the main evolutionary processes contributing to the patterns of neutral spatial genetic structure of populations [9]. Connectivity refers to the movement of individuals between populations [10] and their settlement and contribution to the gene pool of the receiving population (i.e. “migration” in population genetics) [11]. Genetic drift is the stochastic fluctuation of allelic frequencies caused by random variations in the reproductive contribution of individuals [12]. Accurate inferences of both processes at contemporary timescales (i.e. year to decades; [13]) are necessary to adequately define marine conservation strategies [14, 15]. However, contemporary migration has received more attention than contemporary genetic drift (e.g. [12, 16]; see [15]). Indeed, contemporary effective population size (Ne) (the estimator of the contemporary influence of drift) has been neglected in marine conservation planning [15, 16], even though it predicts the populations´ viability and adaptive potential under environmental change [15].

The importance of these evolutionary processes at contemporary timescales calls for a better understanding of their interactions with ecological characteristics of the populations (e.g. demographic traits). The value of the combination of ecological and evolutionary data for the conservation of biodiversity has been acknowledged in recent times [17, 18]. Indeed, several studies done in the terrestrial and freshwater environments have proven the reciprocal influence between evolutionary and population dynamics processes (e.g. between migration and population growth; [19–21]), and the influence of demography on genetic structure has been widely explored (e.g. [22], in plants; [23], in snails; and [24], in fish). However, this approach is seldom applied in the marine realm (but see [25, 26, 27]) and it is still scarcely used in marine conservation planning (e.g. MPAs) (but see [27]). Moreover, none of these studies has focused on habitat-forming organisms, despite their importance for marine conservation.

Previous demographic (e.g. [28–30]) and population genetics (e.g. [31–33]) studies of marine habitat-forming species have greatly contributed to their conservation and should be accounted for in marine conservation planning. However, most of them have been conducted following the classical dichotomy of evolutionary and ecological timescales [34], omitting the eco-evolutionary dynamics [35, 36]. Furthermore, they were mainly centered on the description of patterns, leaving the underlying demographic and genetic processes little explored.

The present study is a first step towards the characterization over a contemporary timescale of the interplay between the demographic traits and the evolutionary processes that underlie the demographic and genetic patterns of the Mediterranean red gorgonian, *Paramuricea clavata*, a sessile and long-lived marine habitat-forming organism of the Mediterranean coralligenous assemblages [37]. Particular emphasis was made on the interaction between contemporary connectivity and genetic drift with the following demographic traits: size distribution, density and mean partial mortality (i.e. partial necrosis of living tissue (tissue injury). The combination of static size distributions with population density is a useful approach to explore population demographic dynamics of sessile, structural organisms, in response to past disturbances [28, 38], while partial mortality is a good demographic indicator of the population's health in clonal organisms such as gorgonians and corals [28, 39, 40].

The main aim of this study was to combine evolutionary and demographic data of a habitat-forming species, for the first time in the marine realm, in order to enhance the decision-making capacities regarding the design of MPAs. This aim was achieved by delving into the populations' evolutionary processes and demographic traits and by exploring the interactions between them. In particular, we (1) examined the patterns of demography and spatial genetic structure (SGS), (2) assessed the underlying processes, with emphasis on contemporary dispersal and genetic drift, (3) characterized the founding of a new population, and (4) explored the interaction of the ecological and genetic processes, shaping these populations. In this study, we highlighted the necessity of accounting for genetic drift in the development of marine conservation measures. We also suggested new perspectives on the functioning and management of low-dispersal marine habitat-forming species.

## Materials and Methods

### Study species

The Mediterranean red gorgonian is a highly vulnerable species from the coralligenous assemblages, one of the richest and most threatened Mediterranean communities [37, 41]. This species is recurrently impacted by large-scale mass mortality events, putatively linked to climate change [41, 42]. It is a gonochoric surface brooder [43], with slow growth (1.8 cm/year) [44, 45] and late sexual maturity, which is attained at an average size of 20 cm (i.e. an approximate age of 13 years) [46]. In general, its populations display low recruitment rates and the prevalence of intermediate size classes (10–30 cm) [28, 47]. This, in addition to its slow population dynamics and low dispersal capacity [33], suggest low resilience to disturbances and low recolonization capacities. In isolated populations (as the ones of this study; see below) this vulnerability is reinforced, as population persistence strongly depends on self-replenishment [48, 49]. Prior population genetics studies on *P*. *clavata* found a significant spatial genetic structure at large scales (∼5 to 3000 Km), produced by a combination between genetic clusters and isolation by distance (IBD) [33]. At local scales (<10 m), no significant spatial genetic structure was observed [50].

### Study area and sampling procedures

**Fig 1:**
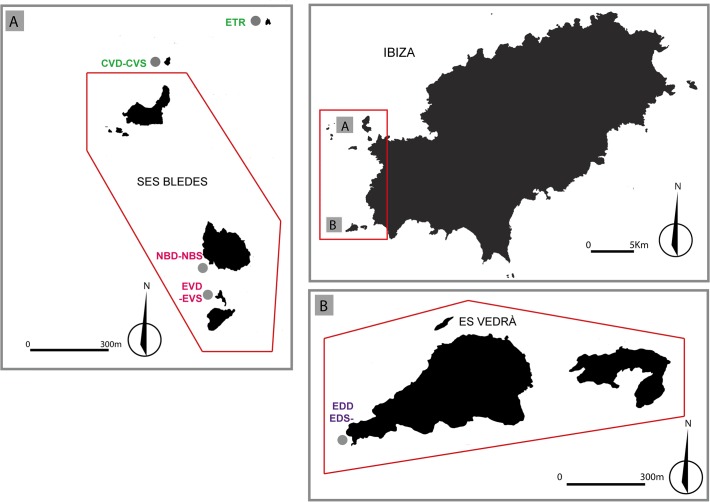
Sampling locations of the populations of *Paramuricea clavata* (grey dots) in Ibiza island. Populations sampled at the same location, but at different depths, are separated by an hyphen. Each population´s name is colored according to their assignment to the Structure clusters (K = 3). The islands inside the red lines in quadrants A and B conform the Nature reserves of Els Illots de Ponent (A), and Es Vedrà and Es Vedranell (B).

Our study took place within the nature reserves of Es Vedrà, Es Vedranell and Els Illots de Ponent, on the west coast of Ibiza (Balearic Islands, Spain), where the possibility of creating a MPA has been discussed. There, populations of *P*. *clavata* only occur in six small islets, above 60 m depth. Samples were taken at two depths, at five of the islets (Fig. 1), except at Escull de Tramuntana, where *P*. *clavata* only occurs at 40–50 m depth (nine populations in total; see Table 1). The population from Escull de Tramuntana, the northernmost islet, is mainly composed by colonies <20 cm, which, considering the slow growth and late sexual maturity of *P*. *clavata* (see above), indicates that it has beenrecently founded. A small apical fragment of 30–50 colonies per site and depth (n = 329; Table 1), was collected by scuba divers and preserved in 95% ethanol at −80°C until DNA extraction. Permits for sampling in Illots de Ponent, Es Vedrà and Es Vedranell Natural Reserves were obtained from “Espais de Natura Balear”, the competent authority. All sampling was performed in accordance with Spanish laws and this study did not involve endangered or protected species.

**Table 1:** Information of the nine sampling populations of *Paramuricea clavata*, listed from North to South.

Location	Population label	Depth (m)	Latitude	Longitude	Sample size
Escull de Tramuntana	ETR	40–55	38°59′6.29″N	1°10′7.12″E	38
Cap Vermell	CVD	45–50	38°58′58.01″N	1°9′41.21″E	36
Cap Vermell	CVS	34	38°58′58.01″N	1°9′41.21″E	44
Na Bosc	NBD	52	38°58′16.96″N	1°9′54.71″E	24
Na Bosc	NBS	36	38°58′16.96″N	1°9′54.71″E	28
Es Vaixell	EVD	45–50	38°58′11.58″N	1°9′56.32″E	27
Es Vaixell	EVS	32–35	38°58′11.58″N	1°9′56.32″E	34
Es Vedrà	EDD	45–50	38°51′45.24″N	1°11′17.14″E	36
Es Vedrà	EDS	30	38°51′45.24″N	1°11′17.14″E	34

### DNA extraction and microsatellite genotyping

Total genomic DNA was extracted using a salting out procedure, following [33]. Individuals were genotyped at seven microsatellite loci: Parcla 09, Parcla 10, Parcla 12, Parcla 14, Parcla 17, PC 3–81 [51], and Par_a [52]. The loci were amplified using the Multiplex PCR Kit (Qiagen) (see S1 Materials and Methods). Genotyping was carried out at the Genotyping and Sequencing facility of Bordeaux (INRA and University of Bordeaux 2) on an ABI 3730 Genetic Analyzer (Applied Biosystems), using GeneScan LIZ 600 (Applied Biosystems) as the internal size standard. Allele scoring was done with the STRand software [53]. The genotyping protocol was validated by extraction, amplification and genotyping of replicates (∼10% of samples). (See S1 Materials and Methods for microsatellites characteristics).

### Demographic and genetic patterns

#### Demographic structure

Demographic patterns were assessed for each population by estimating the population density (n° of colonies/m^2^), the mean partial mortality (i.e. mean extent of injury), the size frequency distribution and the skewness coefficient. Skewness is a statistical measure of a distribution's symmetry; if it is significant, the distribution is asymmetric. Positive skewness indicates the prevalence of small size classes, while negative skewness points to the dominance of large size classes. All parameters were estimated following [28, 54].

#### Genetic diversity

Genetic diversity was explored by calculating (1) the observed heterozygosity (H_o_) and the unbiased expected heterozygosity of Nei (H_e_) [55] with Genetix v.4.05.2 [56], (2) the number of alleles per population (N_a_) with Fstat v.2.9.3.2 [57] and, (3) the allelic richness (A_r_) and private allelic richness (A_p_), using the rarefaction method implemented in HP-Rare [58] with a minimum number of genes equal to 23.

#### Genetic structure

Global and pairwise population genetic differentiation was measured with the θ estimator of F_ST_ [59], computed in Genepop [60]. Significance was tested with the exact test for genic differentiation of Genepop using the default parameters. Additional genetic differentiation was tested by estimating local F_ST_´s (population-specific F_ST_) [61, 62] with Geste v.2.0 [62], using the parameter set described in [63]. Since the computation of local F_ST_ is based on the F-model [64, 65], which accounts for differences in effective sizes and migration rates among populations [61], this parameter can help better describe the genetic structuring of populations. Moreover, it provides useful information about the strength of genetic drift, as it measures “the degree of genetic differentiation between each descendant population and the ancestral population” [62].

The spatial genetic structure was analyzed by testing for isolation by distance (IBD) and genetic clustering. IBD was tested with Genepop, performing a linear regression between pairwise F_ST_/(1-F_ST_) and the logarithm of pairwise geographical distances, as recommended in a 2D model [66]. Pairwise geographical distances were calculated with Google Earth v.7.1.1 (http://earth.google.com) and the significance of the regression was tested with a Mantel test (n = 2000). A Bayesian clustering analysis was made with Structure v.2.3.4 [64, 67] to infer the number of genetic clusters (K). We used the admixture model with correlated allele frequencies [64], and the locprior [68] and recessive allele [69] options. The program was run ten times for each K value ranging from one to nine, with 5x10^5^ iterations and a burn-in period of 5x10^4^. To determine the K that best captured the structure of the sample, we plotted the log probability of the data (LnP(D)) [70] as a function of K across the ten runs. For the chosen K, the results of all the runs were averaged with Clumpp v.1.1.2 [71], and visualized with Distruct v.1.1 [72]. To explore the partitioning of the genetic diversity across the identified clusters (see results), a hierarchical analysis of the molecular variance (AMOVA) was performed in Arlequin v.3.5.1 [73] with 1000 permutations.

### Evolutionary processes

#### Contemporary connectivity and origin of Escull de Tramuntana

Contemporary connectivity is one of the main evolutionary processes shaping the spatial genetic structure of populations [9]. In this study, it was assessed at two complementary timescales: (1) by estimating the average migration rates during the last several generations of individuals (i.e. recent migration rates), and, (2) by detecting first generation migrants, which are the individuals born in a population different from that where they reside [74]. Bayesass 3.0 [75] was used to estimate the interpopulation recent migration rates. It was run five times with different seed values, 25x10^6^ iterations, a burn-in period of 25x10^5^ and a sampling frequency of 2000. Convergence was achieved in all runs and the results were averaged across the five runs. The mixing parameters were adjusted to achieve acceptance rates of 20–40%, following [76]. Geneclass 2.0 [77] was used to detect inter-population first generation migrants. It was run with the L_home likelihood estimation [74], the Bayesian computation of Rannala and Mountain [78] and the Monte Carlo resampling of Cornuet et al. [79] (n = 10000; α = 0.05). To determine the migration processes occurring in the region (i.e. across each genetic cluster), both recent migration rates and first generation migrants were averaged over the populations composing each group. In order to explore the founding of Escull de Tramuntana (a recently established population; see above), the previous analyses were used to determine the origin of the colonies of this islet. Accounting for the isolated nature of our study system, we considered all the other populations to be putative suppliers of immigrants in Escull de Tramuntana.

To gain more insight into the opened/closed nature of clusters and populations, the kinship coefficient of Loiselle [80], which provides an index of relative relatedness between each pair of individuals [81], was computed with Spagedi v.1.3 [82]. Kinship analyses are a valuable complement to infer contemporary connectivity, as they help to elucidate which populations exhibit less genetic exchange, when F_ST_ values are low [83].

#### Genetic drift and migration-drift equilibrium

The strength of genetic drift in each population was determined by estimating the contemporary effective population size (N_e_) of each population. N_e_ was estimated with the single-sample Bayesian computation method implemented in ONeSAMP 1.2 [84] using a minimum prior of 3 and a maximum prior of 300.

To assess if the genetic clusters were at migration-drift equilibrium, the likelihoods of two contrasted models of allele frequencies evolution (i.e. migration-drift equilibrium versus drift) were computed with 2MOD [85]. The program was run with 10^5^ iterations, 10% of which was discarded, as recommended by the author.

### Contemporary interplay of ecology and evolution

To explore the interaction between the evolutionary processes and the demographic traits, three groups of factors were conceived: allelic richness (A_r_), private allelic richness (A_p_) and number of alleles (N_a_) were grouped as genetic factors; recent migration rates, first generation migrants and kinship constituted the group of connectivity factors; and the skewness coefficient, density and partial mortality were grouped as demographic factors. Using these groups of factors, we assessed the interplay of demography and genetics at three different levels. First, the generalized linear model of Geste v.2.0 was used to determine the effect of each group of factors on the genetic structuring (local F_ST_) of populations (see [26]). Then, complementary relations were examined, by correlating each group of factors with local F_ST_´s and N_e_. Finally, associations among groups of factors were explored (e.g. between genetic and connectivity factors). Spearman correlation coefficients for non-parametric variables, with a two-tailed significance test, were used and computed with SPSS 15.0 for Windows®.

The significance of all the multiple tests performed in this study was adjusted with the false discovery rate (FDR) method [86].

## Results

### Demographic and genetic patterns

#### Demographic structure

The size frequency distribution varied in all populations (S1 Fig.). Larger classes (40 to 80 cm) prevailed in two populations (Na Bosc Deep and Es Vaixell Deep). The smallest and non-reproductive colonies (0–10 cm) were predominant in approximately half of the populations. They prevailed in three of the four shallow populations and in Escull de Tramuntana, which was mainly composed of non-reproductive colonies (92% of colonies <20cm). The population density was also highly variable ranging between 6.8 col/m^2^ in Escull de Tramuntana and 57.6 col/m^2^ in Cap Vermell Deep (mean ± SD = 19.2 ± 16.3). Partial mortality varied between 2.2% and 25.2% (13.5% ± 6.8%), being Es Vedrà Deep the population exhibiting more injuries. See Table 2 and S1 Fig. for details.

**Table 2:** Summary statistics of genetic differentiation, drift, genetic diversity, connectivity and demography of the nine populations of *Paramuricea clavata*.

					*Genetic factors*					*Connectivity factors*			*Demographic factors*			
Population	Local F_ST_	95%HPDI	Ne	95%CL	Ar(23)	Ap(23)	Na	Ho	He	Kinship	MR	FGM	D	M	Skewness	(Sig. >2)
ETR	0.015	0.005–0.026	135	88–304	8.710	0.440	10.143	0.755	0.746	0.012	0.325	0.263	6.80	2.24	**2.01**	7.72
CVD	0.043	0.023–0.067	64	45–113	7.230	0.150	8.000	0.694	0.713	0.024	0.254	0.139	57.60	13.78	**1.00**	4.97
CVS	0.030	0.015–0.047	106	70–231	7.740	0.270	8.857	0.743	0.746	0.015	0.167	0.136	11.81	17.26	**1.24**	4.08
NBD	0.012	0.001–0.024	60	42–114	8.800	0.470	8.857	0.788	0.773	0.012	0.323	0.333	11.00	16.64	0.08	0.26
NBS	0.019	0.006–0.034	72	48–153	8.460	0.740	9.000	0.733	0.735	0.024	0.257	0.250	8.38	7.27	**1.14**	3.18
EVD	0.020	0.006–0.037	62	43–121	7.850	0.160	8.286	0.703	0.738	0.007	0.242	0.444	11.75	13.51	0.49	1.96
EVS	0.020	0.008–0.032	113	74–258	8.770	0.800	9.714	0.779	0.761	0.024	0.325	0.206	22.86	8.08	**0.86**	3.89
EDD	0.093	0.060–0.129	50	36–92	6.930	0.130	7.714	0.818	0.769	0.075	0.238	0.139	11.79	25.22	**0.59**	2.61
EDS	0.081	0.050–0.113	54	41–106	7.280	0.230	7.857	0.769	0.738	0.079	0.158	0.118	31.09	17.81	**1.12**	6.06
Mean	0.037		79		7.974	0.377	8.714	0.754	0.746	0.030	0.254	0225	19.23	13.53	0.95	

Local F_ST_: population-specific F_ST_s (Foll and Gaggiotti, 2006); 95%HPDI: highest probability density interval of local F_ST_; Ne: effective population size; 95%CL: 95% confidence limit of N_e_; Ar(23) and Ap(23): allelic and private allelic richness (rarefaction size = 23 genes); Na: mean number of alleles per population; Ho: observed heterozygosity; He: expected heterozygosity (Nei, 1973); Kinship: kinship coefficient (Loiselle et al., 1995); MR: proportion of recent migration rates; FGM: proportion of first generation migrants; D: population density; M: mean % of injured surface; Skewness: skewness coefficient (significant coefficients are in bold).

#### Genetic diversity

Observed heterozygosity (H_o_) varied from 0.694 to 0.818 with a mean value of 0.754, while expected heterozygosity (H_e_) ranged between 0.713 and 0.773 and presented a mean value of 0.746. Escull de Tramuntana displayed the highest number of alleles (10.14), while Es Vedrà Deep presented the lowest (7.71). The mean values of allelic richness (A_r_), private allelic richness (A_p_) and number of alleles (N_a_) per population were 7.97, 0.38 and 9, respectively. Es Vedrà Deep showed the lowest A_r_ and A_p_ (6.93 and 0.13, respectively), while Na Bosc Deep presented the highest A_r_ (8.80) and Es Vaixell Shallow the highest A_p_ (0.80). See Table 2.

#### Genetic structure

**Fig 2:**
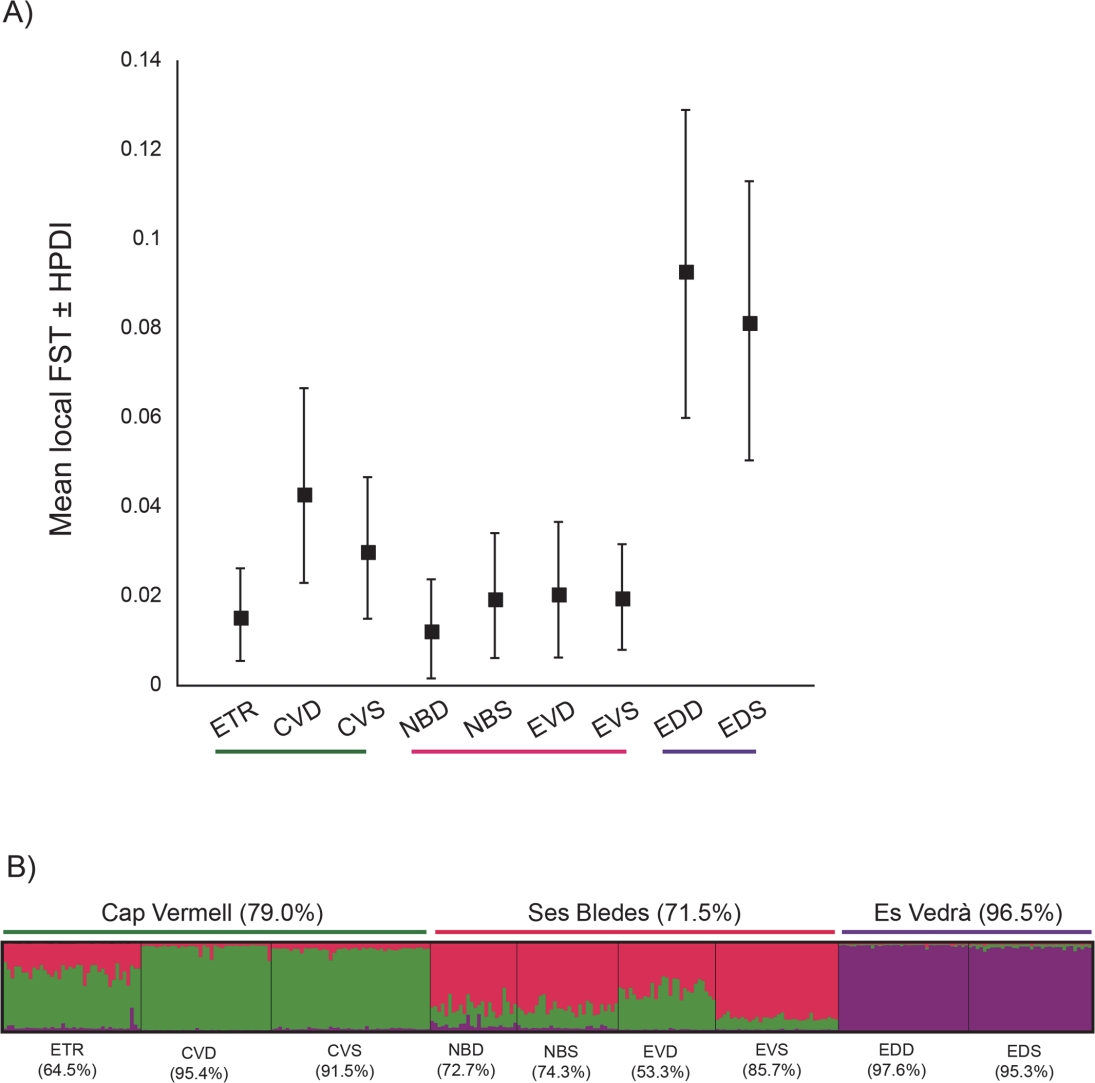
Population structure of *Paramuricea clavata* in Ibiza. (A) Local F_ST_s per population as computed by Geste v.2.0. Filled squares represent the mean value and bars represent the upper and lower limit of the 95% high probability density interval (95% HPDI). The colored bars below the populations´ labels indicate the cluster to which they were assigned in Structure (see Fig. 2B). (B) Clustered structure as revealed by Structure. Each individual is represented by a vertical line, where the different color segments indicate the individual proportion of membership to each cluster (K = 3). Each population is delineated by black vertical lines and labeled as in Table 1. The mean assignment % of each population to the correspondent cluster is given in brackets below the population´s label. The mean assignment % per cluster is given in brackets after the cluster´s name.

Global population differentiation was low, but significant (F_ST_ = 0.035; P < 0.001). Pairwise F_ST_´s ranged from −0.002 to 0.077 and all but four pairwise comparisons (36 comparisons in total) were significant after FDR correction (Na Bosc Deep (NBD) vs. Escull de Tramuntana, NBD vs. Na Bosc Shallow, NBD vs. Es Vaixell Deep, and Cap Vermell Deep vs. Cap Vermell Shallow; see S1 Table). The analysis of local F_ST_s showed that the populations from Es Vedrà were the most differentiated from the others, as they displayed the highest local F_ST_ values (Es Vedrà Deep: 0.093, 95%HPDI: 0.0599–0.129; Es Vedrà Shallow: 0.081, 95%HPDI: 0.0504–0.113) (Fig. 2A, Table 2). On the contrary, Na Bosc Deep was the least differentiated population (local F_ST_ of 0.011 and 95%HPDI = 0.002–0.024).

A significant pattern of isolation by distance (IBD) was detected (R^2^ = 0.72; P = 0.001) (S2 Fig.). The Bayesian clustering analysis identified three genetic clusters (K = 3) (Fig. 2B): Cap Vermell Deep and Cap Vermell Shallow composed the Cap Vermell cluster; Na Bosc Shallow, Na Bosc Deep, Es Vaixell Deep and Es Vaixell Shallow were grouped into the Ses Bledes cluster, although Es Vaixell Deep was similarly assigned to the Ses Bledes and Cap Vermell clusters (53.3% vs. 45.7%; Fig. 2B); and Es Vedrà Deep and Es Vedrà Shallow formed the Es Vedrà cluster. The individuals from Escull de Tramuntana were assigned to the Cap Vermell cluster (Fig. 2B). The AMOVA supported the clustering structure (differences among groups: 3.56%, p<0.005) (S2 Table).

### Evolutionary processes

#### Contemporary connectivity and origin of Escull de Tramuntana

**Fig 3:**
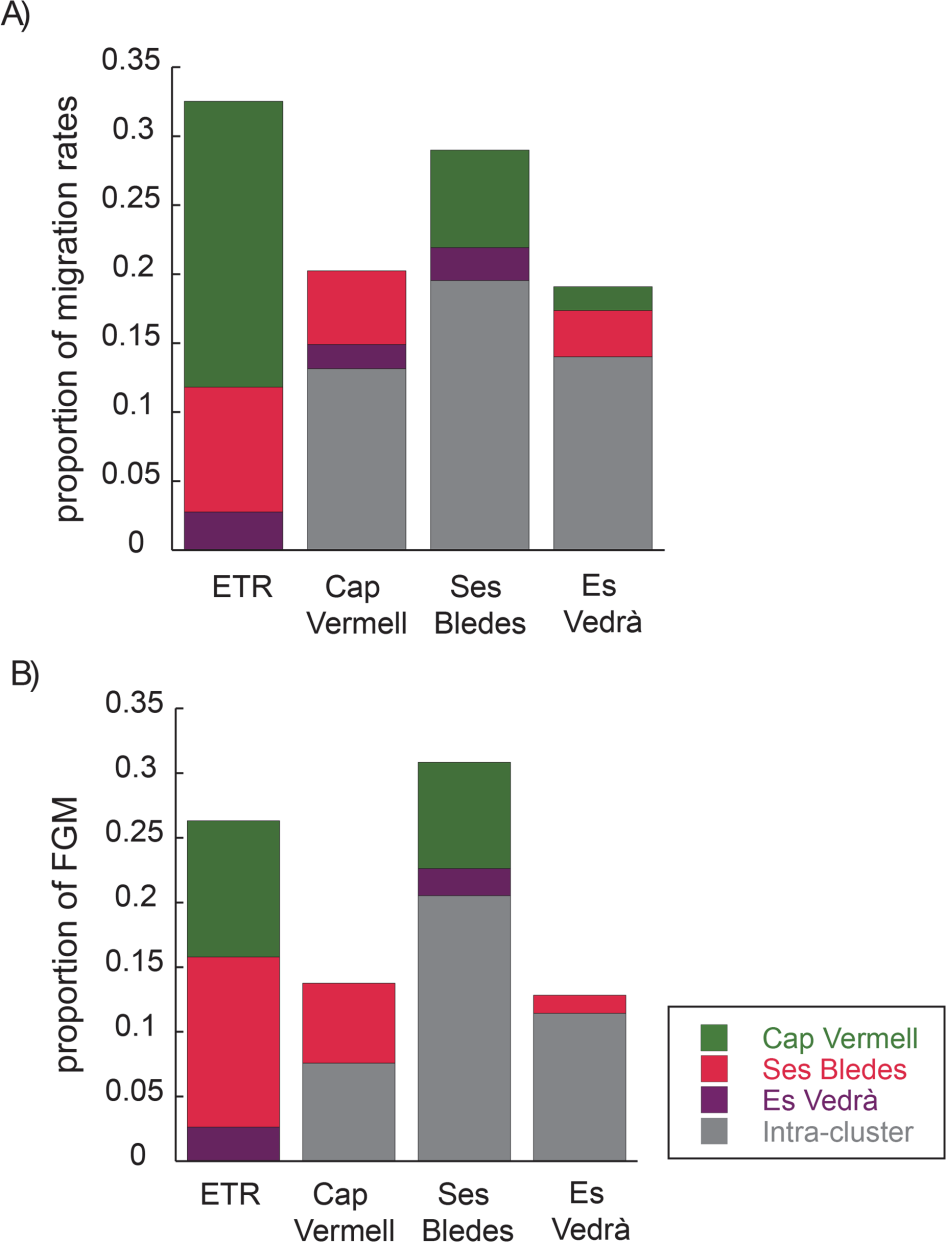
Migration patterns of *Paramuricea clavata* in Ibiza. In both plots each bar represent the proportion of immigration in ETR and each cluster. Since one of our objectives was to determine the origin of ETR, this population was left apart from Cap Vermell cluster. Green, fucsia and purple indicate the proportion of immigration originated in Cap Vermell, Ses Bledes or Es Vedrà clusters, respectively (inter-cluster immigration). Gray indicates the proportion of immigration coming from populations belonging to the same cluster (intra-cluster immigration). (A) Proportion of recent migration rates as computed by Bayesass 3.0, and (B) proportion of first generation migrants (FGM) detected by Geneclass 2.0.

The analysis of migration rates and first generation migrants among clusters revealed a higher intra-cluster migration than inter-cluster migration in the three genetic groups (Fig. 3; S3 Table). The more closed clusters detected by Structure (Cap Vermell and Es Vedrà clusters, Fig. 2B) were indeed, the more closed in terms of connectivity (i.e. receiving less immigration). For both groups, most of the immigration originated from Ses Bledes, while the main immigration source for this latter group was the Cap Vermell cluster (Fig. 3A; S3 Table). The analysis of first generation migrants showed highly similar results (Fig. 3B; S3 Table). The analysis of kinship coefficients confirmed the closed nature of the Es Vedrà cluster, as its populations displayed the highest values (0.092 for Es Vedrà Shallow and 0.088 for Es Vedrà Deep; Table 2). On the contrary, Escull de Tramuntana exhibited the lowest value (0.024), indicating it is a population with more genetic exchange than the others. According to the recent migration rates analysis, Cap Vermell cluster was responsible for 63.7% of the immigration in Escull de Tramuntana, whereas Ses Bledes and Es Vedrà clusters contributed 27.8% and 8.5%, respectively (Fig. 3A; S3 Table). The first generation migrants analysis revealed that the Cap Vermell cluster contributed 40% of the immigrants, while Ses Bledes cluster was the origin of 50% of the migrants. The remaining 10% (i.e. one individual) originated in Es Vedrà (Fig. 3B; S3 Table).

#### Genetic drift and migration-drift equilibrium

Genetic drift was acting upon the most isolated populations (i.e. Es Vedrà Shallow and Deep) confirming the results of the connectivity analyses. In the Es Vedrà cluster, the support for the drift model in the tests for migration-drift equilibrium was 93.6%, while the Cap Vermell and the Ses Bledes clusters were in gene flow-drift equilibrium, with a support of 79.9% and 95%, respectively. Moreover, the populations from the Es Vedrà cluster showed the lowest N_e_ (50 and 54 for Es Vedrà Deep and Es Vedrà Shallow, respectively), in accordance with their high local F_ST_ values (see above). On the contrary, Escull de Tramuntana exhibited the highest N_e_ (135), in accordance with the higher gene flow occurring at this islet (see Table 2).

### Contemporary interplay of ecology and evolution

The generalized linear models revealed that the genetic structure was most related to allelic richness (A_r_), kinship and partial mortality, from the genetic, connectivity and demographic factors, respectively (Table 3). The Spearman correlations showed that local F_ST_ was strongly and negatively associated to allelic richness (A_r_), private allelic richness (A_p_) and number of alleles (N_a_), from the genetic factors and with first generation migrants and recent migration rates, from the connectivity factors. Local F_ST_ was also strongly and positively associated to kinship. Effective population size (N_e_) was strongly and positively correlated with number of alleles (N_a_) from the genetic factors, and negatively associated with partial mortality from the demographic factors. The correlations between groups of factors revealed an interaction between the genetic and the demographic groups, through the negative association of number of alleles (N_a_) with partial mortality and between the connectivity and the genetic groups of factors through the positive association of migration rates with allelic richness (A_r_) and number of alleles (N_a_). Interactions were also observed between the demographic and the connectivity groups through the negative relation of partial mortality and migration rates (Table 4).

**Table 3:** Contemporary interplay of ecology and evolution.

	Factor	Sum of posterior probabilities
*Genetic factors*	Ar	**0.874**
	Ap	0.196
	Na	0.190
*Connectivity factors*	Kinship	**0.677**
	MR	0.352
	FGM	0.132
*Demographic factors*	D	0.174
	M	**0.803**
	Skewness	0.227

The influence of genetic, connectivity and demographic factors on genetic structuring (local F_ST_) is presented as the sum of posterior probabilities computed by Geste v.2.0.

The value of the factor with the highest score within each group is in bold.

**Table 4:** Contemporary interplay of ecology and evolution. Spearman´s correlation coefficients (ρ).

				*Genetic factors*			*Connectivity factors*			*Demographic factors*		
		Local F_ST_	Ne	Ar	Ap	Na	Kinship	MR	FGM	D	M	Skewness
*Genetic factors*	**Ar**	**−0.967**	0.500	-								
	**Ap**	**−0.783**	0.600	**0.867**	-							
	**Na**	**−0.845**	**0.887**	**0.812**	**0.837**	-						
*Connectivity factors*	**Kinship**	**0.733**	−0.383	−*0*.*670*	−0.317	−0.536	-					
	**MR**	**−0.870**	0.617	**0.733**	0.454	**0.795**	−0.467	-				
	**FGM**	**−0.720**	0.184	0.644	0.293	0.454	**−0.812**	0.653	-			
*Demographic factors*	**D**	*0*.*667*	−0.350	−0.583	−0.433	−0.619	0.633	−0.500	−*0*.*678*	-		
	**M**	*0*.*700*	**−0.783**	−0.617	−0.600	**−0.845**	0.467	**−0.800**	−0.603	0.550	-	
	**Skewness**	−0.201	0.617	0.050	0.252	0.410	−0.217	0.017	−0.402	−0.200	−0.333	-

Significant correlations after FDR are in bold and significant correlations before FDR are in italic (P<0.05). Factors´ labels as in Table 2.

## Discussion

### Spatial heterogeneity in demography and genetics: from patterns to processes

The populations of *Paramuricea clavata* from Ibiza exhibited a heterogeneous demographic structure at two different spatial scales (<30 m and <14 Km). At the smallest scale, juveniles and small colonies prevailed in the shallower populations, whereas large colonies dominated in the deeper populations indicating a more mature stage of development [82]. The coexistence of both juvenile and large colonies, a pattern that was previously reported for healthy populations of this species [28] and other gorgonians (see [87] for examples) suggest that, in Ibiza, the populations of *P*. *clavata* are “demographically healthy”. At larger scales, the demographic heterogeneity was supported by the high variation of partial mortality and density across all populations.

A highly heterogeneous genetic structure was also observed at the small and large spatial scales. In general, pairwise F_ST_ values were low, but significant. At the more local scale (∼30 m), some pairwise comparisons were significant (i.e. Es Vaixell Deep-Es Vaixell Shallow, Es Vedrà Deep-Es Vedrà Shallow), while others were not (i.e. Cap Vermell Deep-Cap Vermell Shallow, Na Bosc Deep-Na Bosc Shallow). Furthermore, the variable local F_ST_s, highlight important variations in the genetic structuring as they indicate how similar each population is compared to the whole set of populations [61]. At the regional level, a combination of a pattern of isolation by distance and regional clusters was revealed. This is in accordance with previous findings for the red gorgonian [33] and other Mediterranean species, such as *Corallium rubrum* [31, 88], and confirms that geographic distance is an important factor molding genetic differentiation. Barriers to gene flow also explain the genetic structure of the studied area, as suggested by the clustering analyses and the AMOVA [89]. Regarding the genetic diversity, the global values of observed heterozygosity (H_o_), expected heterozygosty (H_e_), number of alleles (N_a_) and allelic richness (A_r_) were similar to those reported for this species at a much larger spatial scale [33]. These high levels of genetic diversity suggest that the populations of *P*. *clavata* from Ibiza constitute a valuable repository of genetic variation that should be conserved [49].

In accordance with demographic and genetic structure patterns, the underlying processes were also spatially heterogeneous. To our knowledge, the spatial variability of the neutral evolutionary processes (drift and migration) on the patterns of genetic structure has been scarcely documented in marine species (but see [26, 83, 90]), despite its implications in MPAs design (see below). To date, the studies of the processes driving the genetic structure of *P*. *clavata* were mainly focused on gene flow. Low levels of gene flow between populations [33] and a short effective larval dispersal [50] were demonstrated. Our study refines these results by expanding the characterization of migration over a contemporary timescale and by focusing on the counter-balancing impact of genetic drift. The migration analyses demonstrated that connectivity among clusters was weak and mainly occurred between neighboring clusters. In all groups, the intra-cluster migration was two to three times higher than the inter-cluster migration. Despite this apparent homogeneity, a spatial heterogeneity in the levels of gene flow was detected. For instance, the Es Vedrà cluster received two times less migration than Ses Bledes cluster. Moreover, the negative association of local F_ST_s with recent migration rates and first generation migrants confirms that contemporary connectivity is a relevant process in the genetic structuring of *P*. *clavata*.

Genetic drift also had a pronounced impact on the genetic structure, as confirmed by the limited N_e_ of populations when compared to the census population size (hundreds to thousands of colonies; Linares C, pers. obs.). These results contrast with the rather large N_e_ reported in *P*. *clavata* using paternity analyses at a small spatial scale (2 m^2^) [50]. Nevertheless, they are concordant with the low values documented for various marine species (see [15, 91] for reviews). Whether effective sizes are small or large in the sea, it is still a matter of debate [16] and complementary studies based on more populations, comparing different methods of estimation (see [92]) should be undertaken to confirm our results. A spatial heterogeneity in the impact of drift was also evidenced at the geographic scale of our study (<14 Km). As an example, local F_ST_s were significantly and negatively related with genetic diversity (e.g allelic richness) and significantly and positively associated with kinship, indicating that the more distinct populations are more prone to the effect of drift and inbreeding [12]. Accordingly, the Es Vedrà cluster, which displayed the highest values of kinship and local F_ST_, is evolving purely through drift, contrary to the two other clusters that are at migration-drift equilibrium.

### Founding of a new population: colonization of Escull de Tramuntana

Colonization processes in *P*. *clavata* remain poorly studied despite their importance in the current environmental context. Our demographic data suggest that Escull de Tramuntana was recently founded, allowing us a first insight into this process. The founding of a new population may be accompanied by a founder effect, which occurs when populations are established from few individuals [93]. It is characterized by genetic drift, changes on allelic frequencies and decreases of genetic diversity [9]. Surprisingly, Escull de Tramuntana was not submitted to more genetic drift than the other populations under study, as revealed by the high N_e_ and low local F_ST_. Moreover, it shows a small kinship coefficient, a high genetic diversity, and a larger amount of immigration when compared to other populations, suggesting that it has been founded from immigrants coming from various genetically differentiated populations. This hypothesis is supported by the origin of the immigrants, which are native to the Cap Vermell, Ses Bledes and Es Vedrà clusters, indicating that a founder effect is not occurring in this population. Complementary studies involving other recently founded populations are needed to generalize this lack of founder effect during the colonization process of *P*. *clavata*. It would be interesting to conduct a temporal survey on the genetic diversity of Escull de Tramuntana, to test for a putative bottleneck during the future reproduction of the currently non-reproductive colonies.

### Contemporary interplay of ecology and evolution

The characterization of the interaction between ecological and evolutionary processes has been widely studied in land and freshwater environments (e.g. [23]), due to its relevance for conservation biology [18, 36]. In the marine realm it has been scarcely examined and only few studies have been conducted. For instance, [26] disentangled the relative impact of environmental and demographic factors on the genetic structure of the Atlantic herring. Moreover, population genetics and demography were also combined to enhance the conservation of two fish species [27]. However, the present study is one of the first to formally examine this interplay in a habitat-forming species.

Here, the most striking relation between demography and genetics arose from the significant interaction of partial mortality with effective population size (N_e_), number of alleles (N_a_) and migration rates. These results indicate that the less diverse populations undergoing a larger effect of drift and receiving less migration (i.e. the Es Vedrà populations) are the most affected in terms of injured surface. In *P*. *clavata*, partial mortality is caused by multiple stressors, among which environmental disturbances, such as thermal anomalies, play a fundamental role [41]. Fitness and the ability to adapt to environmental change are diminished in populations undergoing a strong drift effect [94] due to the increase of their genetic distinctiveness and the reduction of their genetic diversity [9]. Moreover, they are at greater extinction risk due to inbreeding, stochastic demographic events and loss of adaptive potential [49]. Taking this into account, our results suggest that the populations from Es Vedrà are at risk, as they may be less able to respond to environmental stress than the more connected and diverse populations [95]. Indeed, other studies have proven that the capacity to respond to environmental stressors declines in genetically eroded populations (i.e. under the effects of drift or inbreeding). For instance, [96] showed that the smallest and most isolated populations of *Fucus serratus* were less resilient to high temperatures likely due to the loss of genetic diversity caused by genetic drift.

No significant interactions of skewness or density with other factors were observed. The lack of relation between density and effective population size (N_e_) is remarkable, as it is the opposite of what has been observed in other organisms, such as salmonids and plants, in which high population densities caused reductions in N_e_ [97, 98]. Furthermore, density was positively associated to local F_ST_ and negatively related to first generation migrants before FDR correction. As these relations (or the lack of them) may help to better understand the interactions of demography and genetics, we believe that additional analyses using more samples should be undertaken.

### Implications for conservation and management

We combined demographic and genetic data to designate conservation units within the context of MPAs design using a habitat-forming organism as a model species. In the marine environment, a similar approach was recently used by [27] to establish management units of anadromous alewife and blueback herring. The examination of the processes underlying the demographic and genetic structure of *P*. *clavata* in Ibiza and the assessment of their interaction allowed us to prioritize conservation efforts for populations within the hypothetical future MPA (S3 Fig.). Taking into account the restricted dispersal of the red gorgonian, the MPA should include all the sampled populations, in order to maximize connectivity [14] and minimize the impact of genetic drift in the whole population system. By protecting the whole system, the persistence, genetic diversity, and adaptive potential of populations may be ensured [49, 99]. Three main management units, corresponding to the genetic clusters, should conform the MPA. Within the Cap Vermell cluster, ETR is of high conservation priority, as it is composed of an expanding population with recruits coming from different locations and may entail a future role as a genetic diversity repository [49]. The remaining populations of the cluster (CVD and CVS) are also important since they provide larvae to ETR and, under a context of disturbances, they may act as suppliers of larvae for depleted populations, thereby contributing to region-wide resilience [49, 100]. The populations comprising the Ses Bledes cluster may play the same role as CVD and CVS regarding the regional response to disturbances. Finally, the two populations of the Es Vedrà cluster require urgent measures of conservation given the elevated impact of genetic drift, their high degree of isolation and their high sensitivity to disturbances.

Our study provides new insights for the conservation of *P*. *clavata* by expanding the existing knowledge of the species´ dispersal patterns at small spatial scales, and by delving into its eco-evolutionary dynamics. Although further validation of our results is needed through the inclusion of additional data, our findings have deep implications for the management of *P*. *clavata* and other habitat-forming species with restricted dispersal abilities. Our results may also be directly applied to the design of future isolated MPAs. More broadly, our study (i) reinforces the value of integrating genetic and demographic data in the design of conservation measures, (ii) provides a useful approach for the management and conservation of marine habitat-forming species with low dispersal capacities, and, most importantly, (iii) evidences the relevance of accounting for genetic drift in marine conservation planning, given its impact on populations responses to disturbances.

## Supporting Information

S1 Dataset Individual genotypes of Paramuricea clavata in Ibiza for seven microsatellite loci (n = 301). (XLSX)

S1 Fig Size frequency distributions of nine populations of Paramuricea clavata in Ibiza. (DOCX)

S2 Fig Isolation by distance (IBD). (DOCX)

S3 Fig Conservation units for a future MPA. (DOCX)

S1 Materials and Methods PCR and microsatellite characteristics. (DOCX)

S1 Table Pairwise FST values. (DOCX)

S2 Table Analysis of molecular variance (AMOVA). (DOCX)

S3 Table Regional migration patterns (among ETR, “Cap Vermell”, “Ses Bledes” and “Es Vedrà” clusters). (DOCX)

S4 Table Null allele frequencies (Null) and f estimator of FIS (DOCX)
