# Supplementary figures and images for: Combining Genetic and Demographic Data for the Conservation of a Mediterranean Marine Habitat-Forming Species
Source: PLoS One. 2015 Mar 16;10(3):e0119585. doi: 10.1371/journal.pone.0119585 (PMC4361678; doi:10.1371/journal.pone.0119585)

**Figure S1.** **Size frequency distributions of nine populations of *Paramuricea clavata* in Ibiza.**


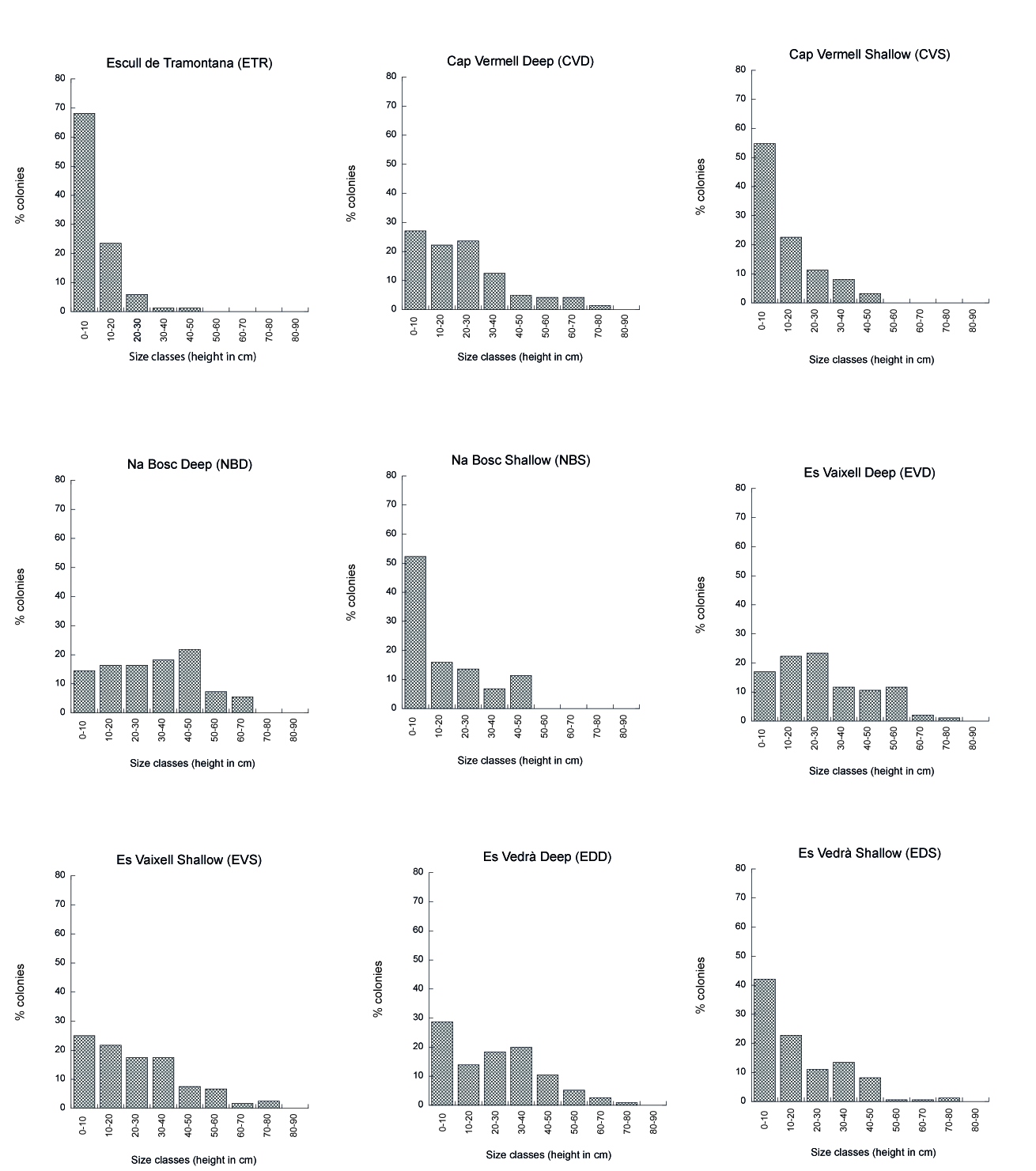

Supplement: S1 Fig — (DOCX) [file pone.0119585.s002.docx]
