# Supplementary material for: Combining Genetic and Demographic Data for the Conservation of a Mediterranean Marine Habitat-Forming Species
Source: PLoS One. 2015 Mar 16;10(3):e0119585. doi: 10.1371/journal.pone.0119585 (PMC4361678; doi:10.1371/journal.pone.0119585)
Supplement: S2 Fig — (DOCX) [file pone.0119585.s003.docx]

**
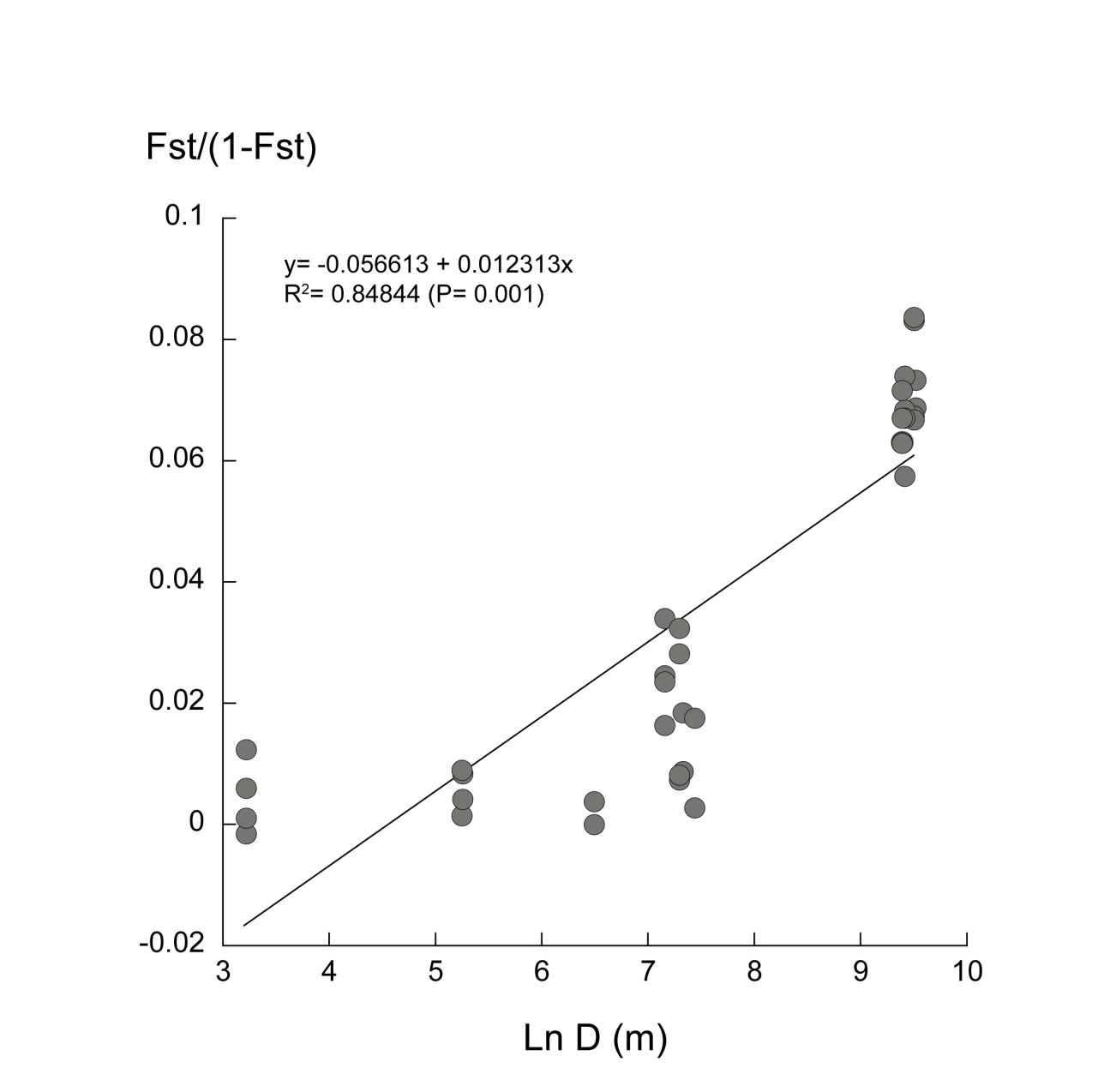
Figure S2. Isolation by distance (IBD).** Correlation between genetic distances (Fs/(1-Fst)) and the logarithm of the geographic distances (m).
