# Supplementary material for: Combining Genetic and Demographic Data for the Conservation of a Mediterranean Marine Habitat-Forming Species
Source: PLoS One. 2015 Mar 16;10(3):e0119585. doi: 10.1371/journal.pone.0119585 (PMC4361678; doi:10.1371/journal.pone.0119585)

**Figure S3.** **Conservation units for a future MPA.** Designated management units for the hypothetical future MPA in the west coast of Ibiza, using the combination of population genetics and demographic analyses.


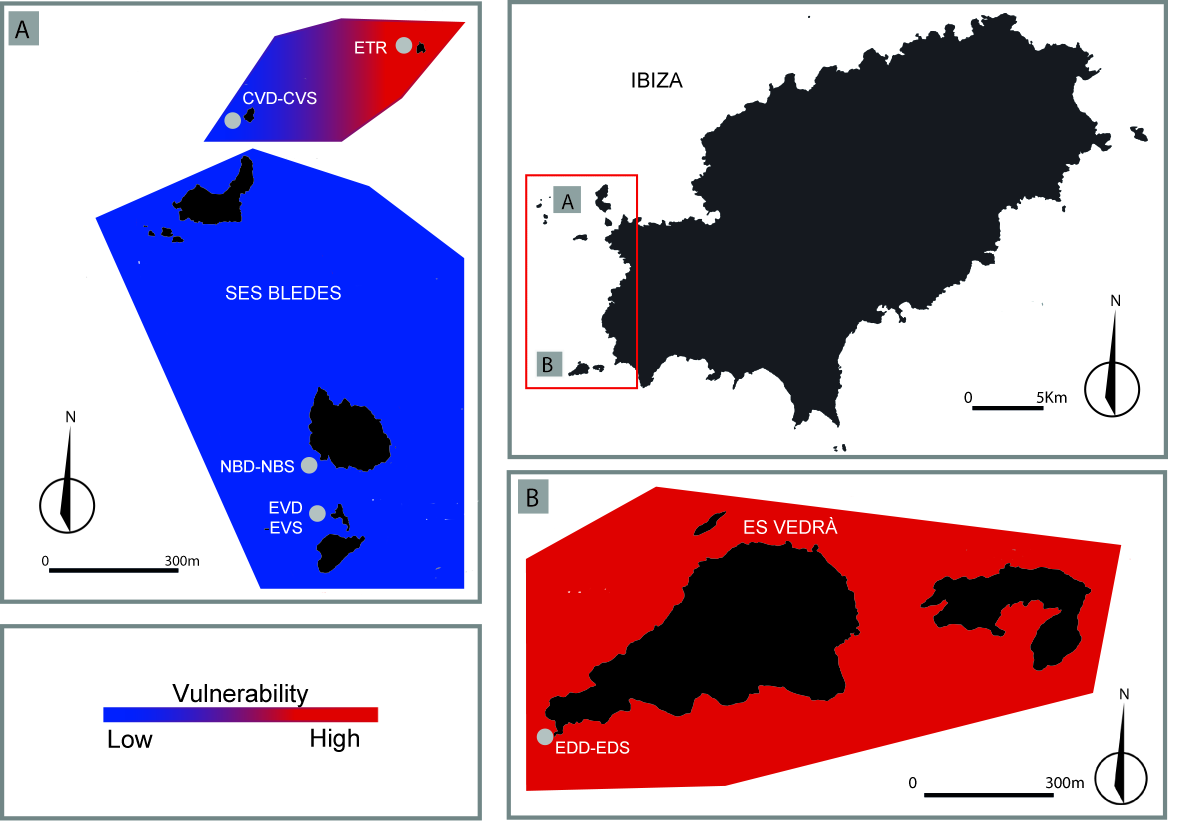

Supplement: S3 Fig — (DOCX) [file pone.0119585.s004.docx]
