# Supplementary material for: Combining Genetic and Demographic Data for the Conservation of a Mediterranean Marine Habitat-Forming Species
Source: PLoS One. 2015 Mar 16;10(3):e0119585. doi: 10.1371/journal.pone.0119585 (PMC4361678; doi:10.1371/journal.pone.0119585)
Supplement: S1 Materials and Methods — (DOCX) [file pone.0119585.s005.docx]

**Materials and Methods**

*PCR characteristics*

The PCR reaction volume was of 11 uL, with 5.5 uL of Master Mix, 1.1 uL of primer mix (with the concentration of primers indicated by the Multiplex Kit instructions), 3.4 uL of sterilized Mili-Q water and 1 uL of DNA. PCRs were performed in either a DNA Engine Peltier Thermal Cycler (Biorad) or a C1000 Thermal Cycler (Biorad), with the following thermal profile: a denaturation step of 15 min at 95 ºC, followed by 30 cycles of denaturation (30 s at 94ºC), annealing (90 s at 59-60 ºC) and elongation (60 s at 72 ºC), with a final extension step of 30 min at 60 ºC.

*Probability of identity, multi-sampled colonies and microsatellite characteristics*

Due to the general spatial arrangement of red gorgonian populations, some colonies may be very close to each other. To determine if there were any multi-sampled individuals in our sample set, we used Gimlet v.1.3.3 [1] to calculate the unbiased probability of identity [2], which is the likelihood that two individuals in the same sample set share the same multilocus genotype by chance, rather than by descent [3], and, to identify unique multilocus genotypes (which in our case correspond to the number of different individuals sampled). After the analyses, a final sample set of 301 individuals was used for the succeeding analyses.

Large allele dropout and scoring errors due to stuttering were tested with Micro-Checker v.2.2.3 [4] using the program´s default values. Null allele frequencies were calculated with Freena [5] following the Expectation Maximization (EM) algorithm of [6], with a bootstrap resampling replicate number of 1000.

Linkage disequilibrium between all pairs of loci within each population was tested with Genetix v.4.05.2 [7] using a permutation procedure (n = 1000). Genepop v.4.2 [8] was used to compute single and multilocus f estimator of the inbreeding coefficient FIS [9] and to test for deviations from Hardy-Weinberg equilibrium, employing the exact tests for heterozygote deficiency or excess, depending on whether the computed f estimator was positive or negative. Significance was assessed by a Markov Chain algorithm [10], using the default parameters.

**Results**

*Probability of identity, multi-sampled individuals and microsatellite characteristics*

The probability of identity in our sample was 1.20x10-08. Twenty-one multilocus genotypes were shared among individuals, each of them being shared between 2 or 3 individuals closely related in space (i.e. sampled in the same location); since they were considered to belong to the same individual, a final sample set of 301 individuals was used for the succeeding analyses.

No large allele dropout or scoring errors due to stuttering were detected. Null allele frequencies varied between 0.001 and 0.063, with a mean frequency over all loci and populations of 0.010 (Table S1). No global linkage disequilibrium (LD) was detected (all P>0.05 after FDR correction), and only two of the 189 tests of local LD (per population) were significant (in NBD, between Par_a and Parcla 10, and in EVD, between Par_a and Parcla 09). Over all loci, no significant heterozygote deficiencies were found. Significant heterozygote excess was detected only for Parcla 10 in EVS. Multilocus values of *f* ranged between -0.064 and 0.049. Considering each locus independently, f values ranged between -0.251 and 0.240. Over all samples and loci, no significant deviations from Hardy-Weinberg equilibrium were detected (Table S4).

**References**

1. Valière N. Gimlet: A computer program for analysing genetic individual identification data. Mol Ecol Notes. 2002; 2: 377-379

2. Kendall M, Stewart A. The Advanced Theory of Statistics, vol. 1. New York: Macmillan; 1977.

3. Ledoux JB, Garrabou J, Bianchimani O, Drap P, Féral JP, Aurelle D. Fine-scale genetic structure and inferences on population biology in the threatened mediterranean red coral, corallium rubrum. Mol Ecol. 2010; 19: 4204-4216.

4. Van Oosterhout C, Hutchinson WF, Wills DPM, Shipley P. Micro-checker: Software for identifying and correcting genotyping errors in microsatellite data. Mol Ecol Notes. 2004; 4: 535-538.

5. Chapuis MP, Estoup A. Microsatellite null alleles and estimation of population differentiation. Mol Biol Evol. 2007; 24: 621-31.

6. Dempster AP, Laird NM, Rubin DB. Maximum likelihood from incomplete data via the EM algorithm. J R Stat Soc Series B. 1977; 39: 1-38.

7. Belkhir K, Borsa P, Chikhi L, Raufaste N, Bonhomme F. GENETIX 4.05, logiciel sous Windows^TM^ pour la génétique des populations. Laboratoire Génome, Populations, Interactions, CNRS UMR 5000, Université de Montpellier II, Montpellier, France. 1996-2004. Available: <http://kimura.univ-montp2.fr/genetix/>. Accessed 17 June 2014.

8. Rousset F. Genepop'007: A complete re-implementation of the Genepop software for Windows and Linux. Mol Ecol Resour. 2008; 8: 103-106.

9. Weir BS, Cockerman CC. Estimating f-statistics for the analysis of population structure. Evolution. 1984; 38: 1358-1370.

10. Guo SW, Thompson EA. Performing the exact test of hardy-weinberg proportion for multiple alleles. Biometrics. 1992; 48: 361-372.
