# Supplementary material for: Combining Genetic and Demographic Data for the Conservation of a Mediterranean Marine Habitat-Forming Species
Source: PLoS One. 2015 Mar 16;10(3):e0119585. doi: 10.1371/journal.pone.0119585 (PMC4361678; doi:10.1371/journal.pone.0119585)
Supplement: S1 Table — (DOCX) [file pone.0119585.s006.docx]

**Table S1. Pairwise Fst values.** Significant pairwise differences, after FDR correction, are in bold.

|  | ETR | CVD | CVS | NBD | NBS | EVD | EVS | EDD | EDS |
| --- | --- | --- | --- | --- | --- | --- | --- | --- | --- |
| ETR | - |  |  |  |  |  |  |  |  |
| CVD | **0.000** | - |  |  |  |  |  |  |  |
| CVS | **0.004** | -0.002 | - |  |  |  |  |  |  |
| NBD | 0.009 | **0.024** | **0.016** | - |  |  |  |  |  |
| NBS | **0.018** | **0.033** | **0.023** | 0.001 | - |  |  |  |  |
| EVD | **0.003** | **0.007** | **0.008** | 0.002 | **0.008** | - |  |  |  |
| EVS | **0.017** | **0.031** | **0.027** | **0.004** | **0.009** | **0.006** | - |  |  |
| EDD | **0.064** | **0.077** | **0.063** | **0.054** | **0.064** | **0.059** | **0.059** | - |  |
| EDS | **0.068** | **0.077** | **0.063** | **0.063** | **0.069** | **0.063** | **0.067** | **0.012** | - |
