# Supplementary material for: Combining Genetic and Demographic Data for the Conservation of a Mediterranean Marine Habitat-Forming Species
Source: PLoS One. 2015 Mar 16;10(3):e0119585. doi: 10.1371/journal.pone.0119585 (PMC4361678; doi:10.1371/journal.pone.0119585)
Supplement: S2 Table — (DOCX) [file pone.0119585.s007.docx]

**Table S2. Analysis of molecular variance (AMOVA).** Samples of *P. clavata* are grouped according to the genetic clusters defined by Structure (K = 3), considering ETR as a separate group.

| Source of variation | d.f. | Percentage of variance | P |
| --- | --- | --- | --- |
| Among groups | 3 | 3.56 | 0.002 |
| Among populations within groups | 5 | 0.50 | 0.007 |
| Among individuals within populations | 593 | 95.94 | 0.000 |
