# Supplementary material for: Combining Genetic and Demographic Data for the Conservation of a Mediterranean Marine Habitat-Forming Species
Source: PLoS One. 2015 Mar 16;10(3):e0119585. doi: 10.1371/journal.pone.0119585 (PMC4361678; doi:10.1371/journal.pone.0119585)
Supplement: S3 Table — (DOCX) [file pone.0119585.s008.docx]

**Table S3. Regional migration patterns (among ETR, "Cap Vermell", "Ses Bledes" and "Es Vedrà" clusters).** Mean percentage ± SD of inter-group immigration rates and their origin are presented. Mean percentage of inter-group first generation migrants ± SD and their origin are presented in brackets.

|  |  | *Origin of immigration (percentage)* | | |
| --- | --- | --- | --- | --- |
| **Recipient group** | **Mean immigration rate**  **(Mean immigrants)** | **"Cap Vermell"** | **"Ses Bledes"** | **"Es Vedrà"** |
| Escull de Tramontana (ETR) | 32.5 (26.3)^*^ | 63.7 (40.0) | 27.8 (50.0) | 8.5 (10.0) |
| Cluster 1 | 7.4 ± 1.0 (6.2 ± 0.9) | - | 76.2 (100.0) | 23.8 (0.0) |
| Cluster 2 | 9.2 ± 4.8 (10.3 ± 9.1) | 73.2 (79.8) | - | 26.8 (20.2) |
| Cluster 3 | 5.2 ± 0.4 (1.4 ± 2.0) | 33.7 (0.0) | 66.3 (100.0) | - |

SD: standard deviation

*These values represent the total (not the mean) immigration rate/proportion of immigrants in ETR
