# Supplementary material for: Combining Genetic and Demographic Data for the Conservation of a Mediterranean Marine Habitat-Forming Species
Source: PLoS One. 2015 Mar 16;10(3):e0119585. doi: 10.1371/journal.pone.0119585 (PMC4361678; doi:10.1371/journal.pone.0119585)
Supplement: S4 Table — (DOCX) [file pone.0119585.s009.docx]

**Table S4. Null allele frequencies (Null) and *f* estimator of Fis.** Significant deviations from Hardy-Weinberg equilibrium, after FDR correction, are in bold.

| Population | Pcla12 | | Pcla14 | | Pcla17 | | Pcla09 | | | Pcla_a | | | Pcla10 | | Pcla81 | | All loci | |
| --- | --- | --- | --- | --- | --- | --- | --- | --- | --- | --- | --- | --- | --- | --- | --- | --- | --- | --- |
|  | *f* | Null | *f* | Null | *f* | Null | *f* | Null | *f* | | Null | *f* | | Null | *f* | Null | | *f* |
| ETR | -0.123 | 0.000 | 0.013 | 0.000 | 0.082 | 0.026 | 0.038 | 0.006 | 0.006 | | 0.000 | -0.027 | | 0.000 | -0.099 | 0.000 | | -0.014 |
| CVD | 0.078 | 0.027 | 0.030 | 0.015 | 0.061 | 0.000 | 0.094 | 0.045 | -0.102 | | 0.000 | 0.036 | | 0.001 | -0.029 | 0.000 | | 0.026 |
| CVS | -0.042 | 0.000 | -0.146 | 0.000 | 0.158 | 0.042 | -0.022 | 0.000 | 0.068 | | 0.041 | 0.130 | | 0.046 | -0.113 | 0.000 | | 0.005 |
| NBD | 0.075 | 0.014 | -0.089 | 0.000 | -0.251 | 0.000 | -0.007 | 0.000 | 0.240 | | 0.051 | -0.109 | | 0.000 | 0.016 | 0.000 | | -0.020 |
| NBS | -0.041 | 0.000 | -0.042 | 0.000 | 0.032 | 0.025 | 0.020 | 0.000 | 0.038 | | 0.000 | -0.072 | | 0.000 | 0.089 | 0.036 | | 0.003 |
| EVD | -0.017 | 0.000 | -0.001 | 0.019 | 0.150 | 0.026 | -0.057 | 0.000 | 0.102 | | 0.034 | 0.122 | | 0.036 | 0.040 | 0.000 | | 0.049 |
| EVS | -0.107 | 0.000 | -0.076 | 0.000 | 0.092 | 0.033 | 0.094 | 0.039 | -0.046 | | 0.000 | **-0.139** | | 0.000 | 0.017 | 0.000 | | -0.025 |
| EDD | -0.020 | 0.000 | -0.117 | 0.000 | -0.185 | 0.000 | 0.038 | 0.000 | -0.008 | | 0.000 | -0.013 | | 0.000 | -0.145 | 0.000 | | -0.064 |
| EDS | 0.111 | 0.063 | -0.031 | 0.000 | 0.036 | 0.023 | -0.109 | 0.000 | -0.174 | | 0.000 | -0.028 | | 0.000 | -0.092 | 0.000 | | -0.043 |
